# Hydrogen Water Drinking Exerts Antifatigue Effects in Chronic Forced Swimming Mice via Antioxidative and Anti-Inflammatory Activities

**DOI:** 10.1155/2018/2571269

**Published:** 2018-04-18

**Authors:** Jesmin Ara, Ailyn Fadriquela, Md Faruk Ahmed, Johny Bajgai, Ma Easter Joy Sajo, Sung Pyo Lee, Tae Su Kim, Jin Young Jung, Cheol Su Kim, Soo-Ki Kim, Kwang Yong Shim, Kyu-Jae Lee

**Affiliations:** ^1^Department of Environmental Medical Biology, Wonju College of Medicine, Yonsei University, Wonju 26426, Republic of Korea; ^2^Department of Global Medical Science, Wonju College of Medicine, Yonsei University, Wonju 26426, Republic of Korea; ^3^Anydoctor Health Care Co. Ltd. 234, Beotkkot-ro, Geumcheon-gu, Seoul 08513, Republic of Korea; ^4^Mymirae Dermatology Clinic and Hydrogen Skin Research Institute, 7, Gukjegeumyung-ro 2-gil, Yeongdeungpo-gu, Seoul, Republic of Korea; ^5^Department of Microbiology, Wonju College of Medicine, Yonsei University, Wonju 26426, Republic of Korea; ^6^Department of Internal Medicine, Wonju College of Medicine, Yonsei University, Wonju 26426, Republic of Korea; ^7^Institute for Poverty Alleviation and International Development, Yonsei University, 1 Yonseidae-gil, Wonju, Gangwon-do 26493, Republic of Korea

## Abstract

**Purpose:**

This study was performed to evaluate antifatigue effect of hydrogen water (HW) drinking in chronic forced exercise mice model.

**Materials and Methods:**

Twelve-week-old C57BL6 female mice were divided into nonstressed normal control (NC) group and stressed group: (purified water/PW-treated group and HW-treated group). Stressed groups were supplied with PW and HW, respectively, ad libitum and forced to swim for the stress induction every day for 4 consecutive weeks. Gross antifatigue effects of HW were assessed by swimming endurance capacity (once weekly for 4 wk), metabolic activities, and immune-redox activities. Metabolic activities such as blood glucose, lactate, glycogen, blood urea nitrogen (BUN), and lactate dehydrogenase (LDH) as well as immune-redox activities such as reactive oxygen species (ROS), nitric oxide (NO), glutathione peroxidase (GPx), catalase, and the related cytokines were evaluated to elucidate underlying mechanism. Blood glucose and lactate were measured at 0 wk (before swimming) and 4 wk (after swimming).

**Results:**

HW group showed a higher swimming endurance capacity (*p* < 0.001) than NC and PW groups. Positive metabolic effects in HW group were revealed by the significant reduction of blood glucose, lactate, and BUN in serum after 4 wk (*p* < 0.01, resp.), as well as the significant increase of liver glycogen (*p* < 0.001) and serum LDH (*p* < 0.05) than PW group. In parallel, redox balance was represented by lower NO in serum (*p* < 0.01) and increased level of GPx in both serum and liver (*p* < 0.05) than PW group. In line, the decreased levels of serum TNF-*α* (*p* < 0.01), IL-6, IL-17, and liver IL-1*β* (*p* < 0.05) in HW group revealed positive cytokine profile compared to PW and NC group.

**Conclusion:**

This study shows antifatigue effects of HW drinking in chronic forced swimming mice via metabolic coordination and immune-redox balance. In that context, drinking HW could be applied to the alternative and safety fluid remedy for chronic fatigue control.

## 1. Introduction

Chronic fatigue is a clinical condition defined by persistent fatigue lasting more than 6 months, which is not amended by rest [1]. Currently, chronic fatigue syndrome (CFS) or idiopathic chronic fatigue has been highly problematic because there is no specific treatment except for lifestyle change, consequently leading to chronic impairment of quality of life [2], and it is also associated with concentration deficiency, memory loss, muscle ache, and sleep deprivation [3]. Moreover, the global incidence of CFS and its prevalence have been steadily rising day by day [3]. Despite the uprising prevalence and incidence of this disease, so far there have been poorly documented studies regarding the effective therapeutics against CFS. With this, most of the patients with this disorder turn to the alternative medicine and other nontraditional therapies [4].

While the pathophysiology of CFS remains unclear, emerging evidences have strongly suggested that the uncoordinated activation of immune system such as inflammation, as well as dysregulation of redox balance and energy metabolism [5], might be a causative factor. Of these potential causes, oxidative/nitrosative stress is the most well-categorized factor [6], consequently inducing the release of proinflammatory cytokines as a secondary response [7]. Previous studies showed elevated levels of proinflammatory cytokines including tumor necrosis factor- (TNF-) *α*, interleukin- (IL-) 1*β*, and IL-6 in affected humans as well as in animal models of chronic fatigue syndrome [8, 9]. Recent studies regarding CFS give emphasis on Th-17 cell mediated proinflammatory cytokines such as IL-23 and IL-17 because of their relation to autoimmune disease and nonspecific inflammation [10, 11]. As a final factor, the disruption of energy homeostasis has been proposed as an alternative etiology for CFS [12]. In this regard, constant or repetitive exercise is prone to deplete glycogen and adenosine triphosphate (ATP), finally leading to systemic exhaustion/fatigue [13]. However, little is known about nonmedicational approach to relieve systemic exhaustion/fatigue. Thus, in the middle of screening nonmedicational fluid against exercise-induced fatigue, we found hydrogen water to be effective in relieving fatigue.

Hydrogen has been well known as a therapeutic antioxidant via selectively scavenging cytotoxic reactive oxygen species (ROS) in tissues [14]. As hydrogen is a gaseous molecule, inhalation might be the most rapid way for scavenging effect. From this point, hydrogen water would be a novel alternative with less adversity than hydrogen gas. Accumulating studies revealed the effectiveness of HW drinking over atherosclerosis, chemotherapeutic nephrotoxicity, and brain injury as well as metabolic syndrome like insulin resistance [14–16]. Previously, we reported redox balance effect of HW in atopic dermatitis and other UV mediated skin injury models [17, 18]. Coupled with these evidences, we hypothesized that HW drinking might be an alternative antifatigue approach against CFS. To address this, we examined antifatigue effect of HW drinking in chronic forced swimming mice via exercise tolerance/swimming endurance capacity, metabolic activities, and immune-redox activities.

## 2. Methods

### 2.1. Animals

Twelve-week-old, specific-pathogen-free, female C57BL6 mice (19-20 g) (Orient Bio Company, Seongnam, Republic of Korea) were used in the experiment. The mice were maintained under controlled conditions (23  ±  2°C, 55  ±  5% humidity, and 12 h light/dark cycle). After a week of acclimation, mice were randomly divided into three groups: nonstressed normal control group (NC, *n* = 7), stressed group treated with purified water (PW, *n* = 7), and stressed group treated with hydrogen water (HW, *n* = 7). PW and HW were supplied ad libitum for consecutive 4 wk and were changed regularly three times a day at an interval of 8 h considering loss of hydrogen molecule from water. To understand the exercise effect of HW in this study, body weight of the mice was measured once weekly for consecutive 4 wk/28 days. The body weight was expressed as percent difference from 0 wk to 4 wk. The animal use and care protocols for this animal experiment were approved by Institutional Animal Care and Use Committee (IACUC), Wonju College of Medicine, Yonsei University, Wonju, Gangwon-do, Republic of Korea. This animal experiment was directed in accordance with the guide for the care and use of laboratory animals published by the US National Institutes of Health (NIH).

### 2.2. Preparation and Standardization of PW and HW

PW was prepared by an electronic purifier, which includes sedimentation filter, pre- and postcarbon filter, and ultrafiltration filter, and was supplied to NC and PW groups. The dissolved hydrogen concentration in PW, used in both NC and PW groups, is 0.0038 ppm measured by dissolved hydrogen meter (MARK-509 Dissolved Hydrogen Meter, Russia). HW (Susosem/hydrogen-enriched water canned drink) was produced by Anydoctor Healthcare Ltd. (Seoul, Republic of Korea) and its producing process was briefly as follows: water was filtered by primary filtration system including activated carbon filter, in which hydrogen gas was continuously supplied with pressure of 0.2 MPa through automatic system equipped with automatic transfer switch and pressure controller in order to highly saturate hydrogen gas into water. Dissolved hydrogen concentration in HW was 1.0–1.2 ppm.

### 2.3. Chronic Stress Induction and Swimming Endurance Capacity

With the exception of the NC group, mice were subjected to chronic fatigue using daily forced swimming test (FST) for 4 wk. Each mouse was dropped into a cylindrical glass swimming pool (18 cm in diameter × 22 cm in height) filled with 10 cm deep water (25 ± 1°C). Mice were placed into each container and allowed to swim for 10 min/d for consecutive 4 wk.

The swimming endurance capacity was measured from latency period to immobile period during swimming. When the mice were dropped in a pool, for first few seconds the mice were immobile which was approximate <3 s which is called the latency period. On the other hand, when the mice exhibited fatigue and the hind legs approximately stopped moving for 10 s, then the immobile period started and mice were removed from water. The maximum mobility time between latency period and immobile period was counted as swimming endurance. Swimming endurance was measured once weekly before the regular stress induction.

### 2.4. Preparation of Serum and Liver Lysate

Mice were sacrificed immediately after swimming at 28th day with standard protocol of anesthesia by isoflurane (Hana Pharm Co., Hwaseong, Republic of Korea). Serum was prepared after collection of the blood from the retro-orbital plexus. Blood was put into BD Microtainer tube (Becton, Dickinson and Company, Franklin Lakes, NJ, USA), allowed to clot for 30 min, and then centrifuged at 14000 rpm for 5 min at 4°C to get the serum. Liver tissue (10 mg) was homogenized in 10% cold RIPA lysis buffer which included proteinase inhibitor (Thermo Scientific, Rockford, USA) using bead milling method (QIAGEN, GmbH, Mannheim, Germany) and centrifuged at 10,000 ×g for 15 min at 4°C, and the supernatant was collected. Samples were stored at −80°C in deep freezer until analysis.

### 2.5. Determination of Blood Glucose and Lactate

The blood samples were collected from caudal vein of mice before swimming at wk 0/day 1 and after swimming at wk 4/day 28. To measure blood glucose and lactate, approximately 15 *μ*L of blood from each mouse was collected by piercing the caudal vein with an insulin syringe. Among the collected 15 *μ*L, 5 *μ*L was used for glucose strip (Roche Accu-Chek Active, Roche Diagnostics GmbH, Mannheim, Germany) and 10 *μ*L was used for lactate strip (Roche Accutrend Plus, Roche Diagnostics GmbH, Mannheim, Germany). Blood glucose and lactate were measured using and by following their company instruction manuals.

### 2.6. Examination of Blood Urea Nitrogen (BUN), Glycogen, and Lactate Dehydrogenase (LDH) Levels in Serum and Liver Lysate

Liver lysate was first normalized for protein concentration using Pierce™ BCA protein assay kit (Thermo Fisher Scientific Inc., Illinois, USA). BUN and LDH activities were measured by a urea nitrogen colorimetric detection kit (Arbor Assays, BioVision Inc., Illinois, USA) and LDH colorimetric assay kit (BioVision Inc., CA, USA), respectively, at 450 nm following the manufacturer's instruction. Glycogen concentration was measured using a glycogen colorimetric assay kit (BioVision Inc., CA, USA) at 570 nm according to manufacturer's manuals.

### 2.7. Examination of Reactive Oxygen Species (ROS) and Nitric Oxide (NO) Levels in Serum and Liver Lysate

ROS in serum and liver lysate were analyzed using 2′,7′-dichlorofluorescin diacetate (DCFH-DA) assay kit (Abcam, Cambridge, USA) according to the instruction manual. In brief, the samples of each group (serum and liver lysate) were incubated with 10 *μ*mol/L of DCFH-DA for 30 min at 37°C in the dark. Then it was read and analyzed using a DTX-880 multimode microplate reader (Beckman Counter Inc., Fullerton, CA, USA) at an excitation wavelength of 488 nm and an emission wavelength of 525 nm.

NO levels were determined using NO detection kit (Intron Biotechnology Company, Sungnam, Korea) according to manufacturer's instruction. Fifty *μ*L of serum and liver lysate was transferred in a 96-well plate and incubated with Reagent A for 10 min and Reagent B for another 10 min at room temperature. The absorbance was read at 540 nm using a multimode microplate reader (Beckman Counter Inc., Fullerton, CA, USA).

### 2.8. Endogenous Antioxidant Enzyme Activities

Glutathione peroxidase (GPx) activities in serum and liver lysate were evaluated by measuring the H_2_O_2_ scavenging capacity using GPx assay kit (Biovision, Milpitas, CA, USA) according to the manufacturer's instruction. In brief, oxidized glutathione, produced upon reduction of H_2_O_2_ by GPx, was recycled in its reduced state by glutathione reductase and reduced nicotinamide adenine dinucleotide phosphate (NADPH). The oxidation of NADPH to NADP^+^ was accompanied by a decrease in absorbance at 340 nm (Beckman Counter Inc., Fullerton, CA, USA).

Catalase activity in serum and liver lysate was measured using catalase colorimetric assay kit (BioVision, Mountain View, CA 94043, USA) according to the instruction manual. Briefly, the rate of decomposition of H_2_O_2_ was measured spectrophotometrically at 570 nm using microplate reader (Beckman Counter Inc., Fullerton, CA, USA). One unit of catalase was defined as the amount of enzyme needed to decompose 1 *μ*M of H_2_O_2_ in 1 min. The catalase activity was normalized to milligram of protein used in the assay and was expressed as mU/ml protein.

### 2.9. Cytokine Analysis

Cytokines such as IL-1*β*, IL-6, IL-17, IL-23, and TNF-*α* in serum and liver were analyzed using a Multiplex Bead Suspension Array Kit (Bio-Rad, San Diego, CA, USA) according to the manufacturer's instructions. The plate was run on a Luminex 200 Bio-Plex Instrument (Bio-Rad, Hercules, CA, USA). Raw data were analyzed by the software using 5-parameter logistic method.

### 2.10. Statistical Analysis

The mean values among the groups were analyzed by GraphPad Prism version 5.0 software packages (GraphPad, La Jolla, CA, USA) and were compared using one-way analysis of variance (ANOVA) followed by subsequent multiple comparison test (Tukey). Differences were considered statistically significant at *p* < 0.05.

## 3. Results

### 3.1. Body Weight and Swimming Endurance

**Figure 1:**
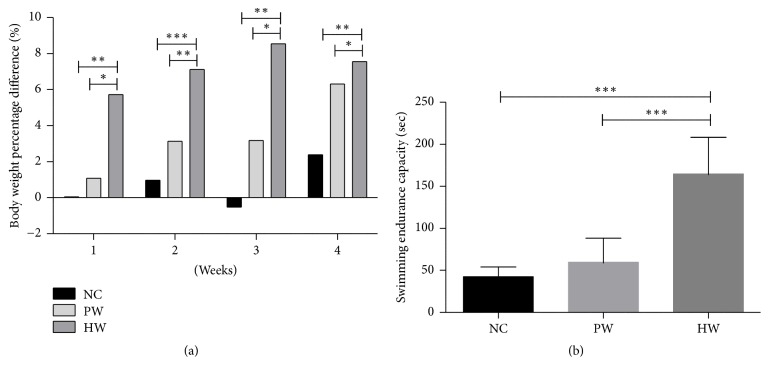
Increasing rate (%) of body weight time-dependently and swimming endurance capacity. Body weight percent difference (a) and swimming endurance capacity (b). Data were expressed as mean ± SD, *n* = 7. ^*∗*^*p* < 0.05, ^*∗∗*^*p* < 0.01, and ^*∗∗∗*^*p* < 0.001. NC: normal control group, PW: stressed group treated with purified water, and HW: stressed group treated with hydrogen water.

Body weight gains of the mice were significantly higher in HW compared to NC and PW group, respectively, during all stages of the experiment for 4 wk (Figure 1(a)). To measure swimming endurance capacity, maximum mobility time was recorded without induction of any external stimulation to all groups of mice once weekly for consecutive 4 wk. After forced swimming stress, maximum mobility time revealed that HW group showed longer swimming endurance time than NC and PW groups (*p* < 0.001, resp.) (Figure 1(b)).

### 3.2. Metabolic Activities (Blood Glucose, Blood Lactate, BUN, Glycogen, and LDH)

**Figure 2:**
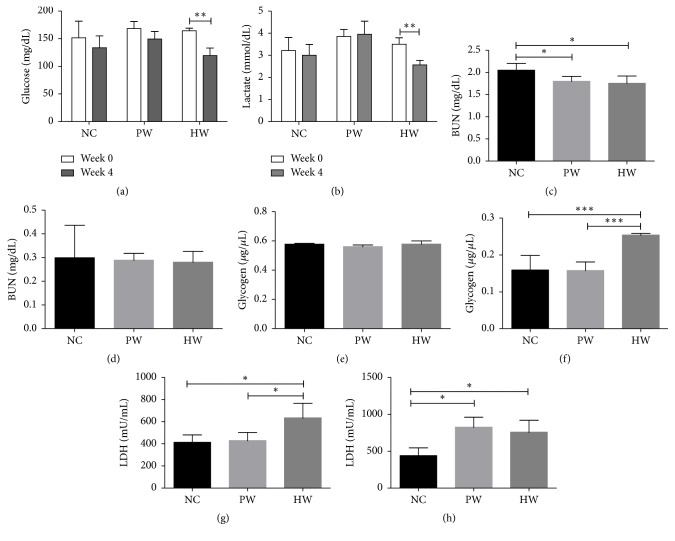
Metabolic activities. Blood glucose (a) and blood lactate (b) levels between wk 0 and wk 4, BUN in serum (c) and liver lysate (d), glycogen in serum (e) and liver lysate (f), and LDH level in serum (g) and liver lysate (h). Data were expressed as mean ± SD, *n* = 7. ^*∗*^*p* < 0.05, ^*∗∗*^*p* < 0.01, and ^*∗∗∗*^*p* < 0.001. NC: normal control group, PW: stressed group treated with purified water, and HW: stressed group treated with hydrogen water.

Forced swimming decreased blood glucose level when comparing before (0 wk) and after experiment (4 wk) in all groups. Notably, only HW drinking significantly decreased glucose level after 4 wk of treatment (*p* < 0.01; Figure 2(a)). Moreover, blood lactate levels also showed significant decrease after 4 wk of HW treatment (*p* < 0.01), while it showed an increased trend in PW group (Figure 2(b)).

BUN level in serum was measured immediately after sacrifice of mice at 28th day, and BUN was significantly reduced in PW and HW groups as compared to NC (*p* < 0.05, resp.; Figure 2(c)), while there was little difference among groups in liver lysate (Figure 2(d)).

Glycogen concentration of HW group was measured after sacrifice of mice at 28th day, and hepatic glycogen was significantly higher (*p* < 0.001, resp.) in HW group than NC and PW groups. By contrast, serum glycogen did not show any significant difference (Figures 2(e) and 2(f)).

LDH level was measured after sacrifice of mice at 28th day. The LDH levels in both serum and liver lysate of HW group were significantly higher (*p* < 0.05, resp.) than those of NC group (Figures 2(g) and 2(h)). Of note, serum LDH level of HW group was significantly higher (*p* < 0.05) than that of PW group (Figure 2(g)).

### 3.3. Redox Balance (ROS, NO, GPx, and Catalase)

**Figure 3:**
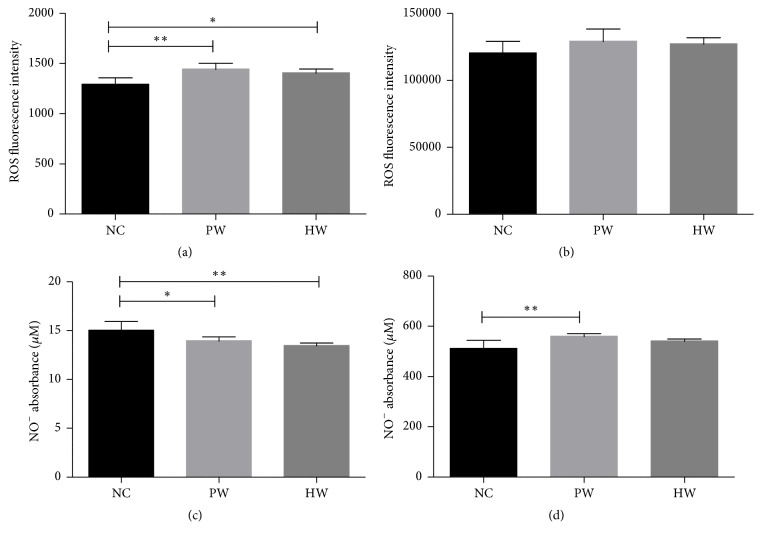
ROS and NO production. ROS level in serum (a) and liver lysate (b) and NO level in serum (c) and liver lysate (d). Data were expressed as mean ± SD, *n* = 7. ^*∗*^*p* < 0.05, ^*∗∗*^*p* < 0.01. NC: normal control group, PW: stressed group treated with purified water, and HW: stressed group treated with hydrogen water.

To gauge oxidative/nitrosative stress, we measured total ROS and NO of both serum and liver lysate. The ROS and NO level were measured immediately after collection of serum and liver lysate after sacrifice of mice at 28th day. First, serum ROS levels in HW and PW groups were higher than that in NC group (*p* < 0.05; *p* < 0.01, resp., Figure 3(a)). In line, hepatic ROS level in all groups showed similar pattern compared to serum ROS (Figure 3(b)).

Second, serum NO levels in PW (*p* < 0.05) and HW groups (*p* < 0.01) were significantly lower than those in NC group (Figure 3(c)). By contrast, hepatic NO levels in PW (*p* < 0.01) group were higher than those in NC group (Figure 3(d)).

**Figure 4:**
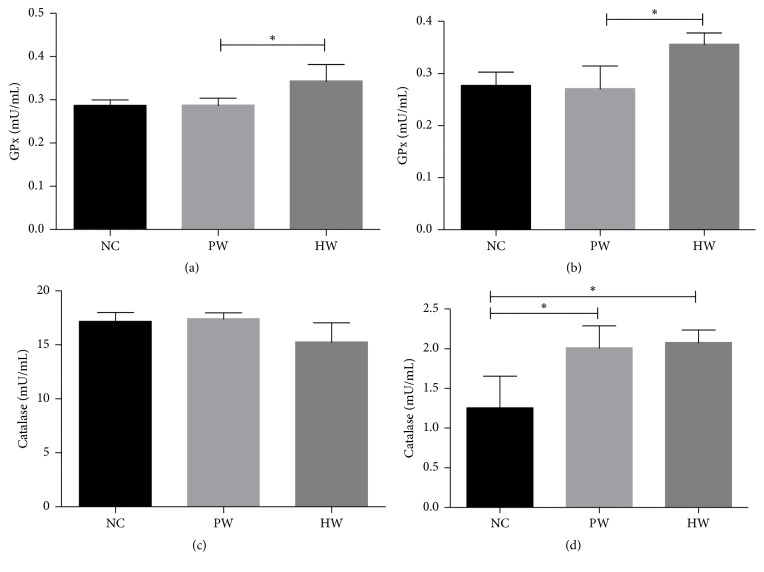
Antioxidant enzyme activities. GPx activity in serum (a) and liver lysate (b) and catalase activity in serum (c) and liver lysate (d). Data were expressed as mean ± SD, *n* = 7. ^*∗*^*p* < 0.05, NC: normal control group, PW: stressed group treated with purified water, and HW: stressed group treated with hydrogen water.

The GPx and catalase concentration were measured at 3rd day after collection of serum and liver lysate. HW administration markedly increased GPx activity compared to NC and PW group in both serum and liver lysate. In particular, serum and hepatic GPx activity of HW group showed significantly higher level than PW group (*p* < 0.05) (Figures 4(a) and 4(b)). Hepatic catalase activity of PW (*p* < 0.05) and HW (*p* < 0.05) groups showed significantly higher level compared to NC group but there was no difference between PW and HW group. However, there was no statistical significance seen in catalase activity in serum among all groups (Figures 4(c) and 4(d)).

### 3.4. The Level of Cytokines

**Figure 5:**
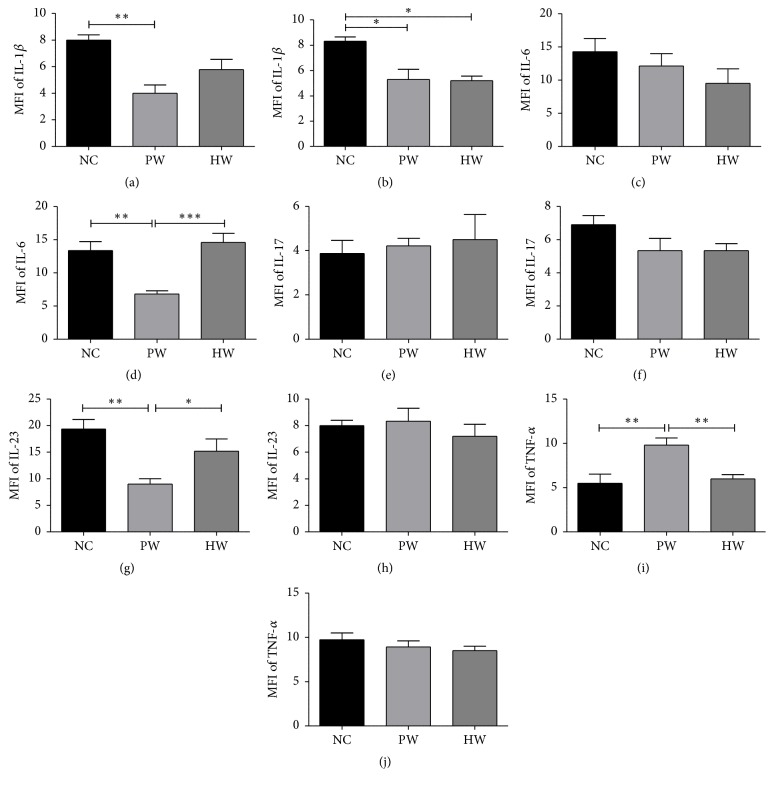
Inflammatory cytokine analysis. IL-1*β* level in serum (a) and liver lysate (b), IL-6 level in serum (c) and liver lysate (d), IL-17 level in serum (e) and liver lysate (f), IL-23 level in serum (g) and liver lysate (h), and TNF-*α* level in serum (i) and liver lysate (j). Data were expressed as mean ± SD, *n* = 7. ^*∗*^*p* < 0.05, ^*∗∗*^*p* < 0.01, and ^*∗∗∗*^*p* < 0.001. NC: normal control group, PW: stressed group treated with purified water, and HW: stressed group treated with hydrogen water.

IL-1*β*, IL-6, IL-23, IL-17, and TNF-*α* were analyzed in both serum and liver lysate at 7th day of sample collection and compared among groups. Serum IL-1*β* level was markedly decreased in PW (*p* < 0.01) and HW groups compared to NC group (Figure 5(a)). In liver lysate, IL-1*β* was significantly decreased in PW (*p* < 0.05) and HW (*p* < 0.05) groups compared to NC group (Figure 5(b)). IL-6 in serum also showed a reduction trend but no statistical significance was observed (Figure 5(c)). However, in liver lysate, IL-6 showed a significant increase (*p* < 0.001) as compared with PW group (Figure 5(d)). Moreover, serum TNF-*α* of HW group showed significantly decreased level as compared to PW group (*p* < 0.01) (Figure 5(i)), while IL-23 and IL-17 in liver lysate did not show significant changes (Figures 5(h) and 5(f)). In addition, serum IL-23 showed significantly higher level in HW group compared to PW group (*p* < 0.05) (Figure 5(g)).

## 4. Discussion

This study showed antifatigue effects of HW drinking in chronic forced exercise mice via metabolic coordination and immune-redox balance. Concretely, this is evidenced by four proofs such as excellent swimming endurance capacity, positive metabolic profile, and immune-redox balance. Of these, we first checked clinical profiles in 4 wk forced swimming mice model, which was well proven for high reproducibility to quantify antifatigue effects [19, 20] because swimming endurance capacity was one of the best indicators for antifatigue effects [19, 20]. Unexpectedly, HW group showed longer swimming endurance time than NC and PW groups (Figure 1(b)). In parallel, significant body weight gain in HW treated mice (Figure 1(a)) throughout 4 wk FST might support the longer swimming endurance since animals with heavier body weight had higher basal metabolism [21], thus enduring bigger work load. Second, to validate this clinical relief, we examined the metabolic activities (blood glucose, blood lactate, BUN, glycogen, and LDH) in FST mice, which were also the relevant biochemical indices to show the magnitude of fatigue [22]. Consistent with this clinical outcome, we found positive metabolic effects in HW group, as evidenced by the significant reduction of blood glucose, lactate, and BUN after 4 wk, as well as the significant increase of liver glycogen and serum LDH. In fact, chronic forced exercise would induce higher level of serum glucose, lactate, and BUN, which may be indicative of activated glycolysis and correlative of protein breakdown, stress, and fatigue, respectively [23], consequently leading to expediting the consumption of the storage glycogen in liver and muscle. To our amazement, only HW drinking clearly reversed these metabolic dysregulation. Increase of liver glycogen and serum LDH might explain this phenomenon. However, the exact molecular switch to coordinate this metabolic imbalance remains to be verified. In CFS, this aberrant metabolic status might be elicited by the dysregulated immune and redox homeostasis.

Toward this, third we measured the levels of redox balance markers such as ROS, NO, GPx, and catalase. It has been well known that endurance exercise or even therapy might induce an increased ROS [24, 25]. In this study, we found that HW could increase the ROS level but less significantly than other stressed groups, even after high endurance, strongly suggesting that HW could reduce the oxidative stress related damage caused by free radicals, thereby protecting the integrity of the liver cell membrane. This could maintain normal physiology of the liver and promote the recovery process after exercise in mice [26]. As another stress marker, NO plays a pivotal role in chronic fatigue related to nitrosative stress because NO is able to react with ROS, thus producing reactive nitrogen species (RNS). These ROS and RNS might aggravate CFS. In this study, HW drinking might dually block oxidative/nitrosative stress path via the decrement of ROS/RNS. This might be partly explained by an increased level of GPx and liver catalase; both enzymes are one of the endogenous cellular defense mechanisms to control ROS [20]. GPx is regarded as one of the first lines of defense of the antioxidant enzyme system against ROS generated during the exhaustive exercise [27]. Catalase is able to catalyse the decomposition of H_2_O_2_ into H_2_O and O_2_ [28]. For instance, previously it was reported that serum GPx activity was decreased with oxidative stress due to exercise-induced fatigue [29]. Of note, higher GPx and catalase activity in HW-fed mice implies that HW might be beneficial against CFS or equivalent fatigue. Taken together, these redox data clearly showed that HW drinking is effective in redox imbalance which incurred in chronic forced swimming mice.

Lastly, we attempted to find immunological evidences. In chronic illness patients, chronic inflammation is commonly observed with huge elevation of proinflammatory cytokines such as IL-1*β*, IL-6, and TNF-*α* [30]. However, in experimental group, the cytokine levels were mostly attenuated by HW therapy. Importantly, IL-6 and IL-23 were involved in maturation procedure of Th-17 cells [31]. In particular, IL-23 is evidenced in counteracting immune suppression, which is known for the key feature of CFS or equivalent setting [32]. In serum, proinflammatory cytokine IL-6 and IL-17 levels were lower in HW than NC and PW groups. By contrast, higher level of IL-23 in NC and HW groups might imply the protective role of IL-23/Th-17 axis in the immunity at mucosal surfaces [33] as well as in the plausible immunosuppression of our fatigue mice model incurred by proinflammatory cytokines.

Collectively, this is completely new* in vivo* experimental research about HW on chronic fatigue mice model and the cumulative records indicated that HW had antifatigue activity, which could evidently extend exhaustive swimming time of mice, inhibit production of blood lactate, decrease BUN, ROS, and NO, and promote the activities of LDH and GPx levels of mice after forced swimming. Liver glycogen was also promoted by HW treatment. However, the key limitation of this research is the absence of muscular system study as chronic fatigue syndrome is closely related to muscular system.

## 5. Conclusion

Taken together, this study shows antifatigue effects of HW drinking in chronic forced exercise mice model via metabolic coordination and immune-redox balance. In that context, HW could be applied to the alternative and safety fluid remedy for chronic fatigue control. Further studies are underway to uncover the molecular mechanism and cytokine networks of HW in this model.

## Abbreviations

ANOVA: One-way analysis of variance
ATP: Adenosine triphosphate
BUN: Blood urea nitrogen
CFS: Chronic fatigue syndrome
DCFH-DA: 2′,7′-Dichlorofluorescin diacetate
FST: Forced swimming test
GPx: Glutathione peroxidase
HW: Hydrogen water
IACUC: Institutional animal care and use committee
LDH: Lactate dehydrogenase
IL: Interleukin
NADPH: Nicotinamide adenine dinucleotide phosphate
NC: Normal control
NIH: National institutes of health
NO: Nitric oxide
PW: Purified water
ROS: Reactive oxygen species
TNF-*α*: Tumor necrosis factor-*α*.
